# Prognostic and functional roles of EIF4G1 in lung squamous cell carcinoma

**DOI:** 10.1007/s13577-023-00884-9

**Published:** 2023-03-10

**Authors:** Baoxin Bai, Lin Dong, Minghao Feng, Zhiwen Zhang, Ying Lu, Zengguang Xu, Yali Liu

**Affiliations:** 1 Research Center for Translational Medicine, Shanghai East Hospital, Tongji University School of Medicine, Shanghai, 200092 China; 2 Research Center for Translational Medicine, Shanghai East Hospital, GuiLin University School of Medicine, Guilin, 541004 China; 3 Hubei University of Medicine, No. 30, Renmin South Road, Maojian District, Shiyan, 442000 China; 4 Department of Cardiothoracic Surgery, Shanghai East Hospital, Tongji University School of Medicine, Shanghai, 200120 China

**Keywords:** Prognosis, EIF4G1, LSCC, Functional role

## Abstract

Eukaryotic translation initiation factor 4 gamma 1 (EIF4G1) is highly expressed in many cancers and affects their occurrence and development. However, the effect of EIF4G1 on the prognosis, biological function and the relevant mechanism in lung squamous cell carcinoma (LSCC) is unclear. Through clinical cases, Cox’s proportional hazard model and Kaplan–Meier plotter survival analysis, we find the expression levels of EIF4G1 are dependent on age and clinical stage, high expression of EIF4G1 could be used to predict the overall survival of LSCC patients. LSCC cell line NCI-H1703, NCI-H226 and SK-MES-1infected with EIF4G1 siRNA are used to detect the function of EIF4G1 with cell proliferation and tumorigenesis in vivo and vitro. The data show that EIF4G1 promotes tumor cell proliferation and the G1/S transition of cell cycle in LSCC, then the biological function of LSCC is effected by the AKT/mTOR pathway. Above all, these results have demonstrated that EIF4G1 promotes LSCC cell proliferation and may represent an indicator of prognosis in LSCC.

## Introduction

LSCC is a specific type of lung cancer. Approximately 20% of lung cancers are LSCC [1]. Although the 5 year overall survival rate of patients with primary LSCC is approximately 37.2% [2], the majority of the patients with LSCC (staging IV) have shown survival rates below 2% [3]. The survival rate of LSCC in the early stage of the disease is much higher than that noted in the advanced stage. LSCC is closely associated with smoking. Carcinogens come in direct contact with respiratory mucosal epithelium, causing squamous epithelial metaplasia, dysplasia and carcinoma in situ to form LSCC [4]. Current research suggests that changes in gene expression and signaling pathways promote the development of LSCC. However, the pathological mechanism of LSCC is not fully understood.

EIF4G1 is overexpressed in bladder, breast, cervical, colon, endometrial, esophageal, kidney, lung, ovarian, pancreatic, head and neck, stomach and testicular cancers [5]. Previous studies have shown a lower survival rate of patients with a higher expression of EIF4G1 [6–8]. EIF4G1 also plays an important role in cancer development. Overexpression of EIF4G1 promotes the proliferation, invasion, migration, apoptosis and tumorigenesis of cancer cells [9–11]. Concomitantly, EIF4G1 expression is associated with the PI3K-AKT pathway in colorectal cancer [7] and it has been shown that SBI-756 blocks EIF4G1 function and inhibits AKT/mTORC1 signaling in melanoma cancers [12]. EIF4G1 is the effector of mTOR [13]. In LSCC, EIF4G1 is located in 3q26-27 [14] and is overexpressed [15]. However, the potential of aberrant EIF4G1 expression to predict LSCC prognosis has not been previously reported. The current study aimed to predict the prognosis and function of EIF4G1 in LSCC.

## Materials and methods

### Tissue samples, immunohistochemical staining and scoring

Tissue arrays and immunohistochemical staining kits were from SHANGHAI OUT BIOTECH CO., LTD. The LSCC HLug-Squ150Sur-02 and HLugS180Sur01 tissue arrays contained clinical specimens from 75 and 90 patients, respectively. A total of 137 pairs were obtained following removal of repetitive and damaged tissues. A total of 137 pairs of LSCC (T) and adjacent tissues (N) were obtained by removing duplicates. The application of the densito quantitative software was used in Quant Center to automatically identify and set all dark brown on the tissue section as strongly positive (3), yellowish brown as moderately positive (2), light yellow as weak positive (1) and blue as negative (0) [16]. Furthermore, each tissue point was identified and analyzed for strong positive, moderately positive, weakly positive and negative areas (unit: pixels). The percentage of positive areas and the H-score were also assessed. The H-score was calculated as follows: H-score = percentage of cells with weak intensity × 1 + percentage of cells with moderate intensity × 2 + percentage of cells with strong intensity × 3 [17].

### Cell culture

The normal human fetal lung fibroblast cell line HFL1 and the human LSCC cell line NCI-H226 was obtained from the Shanghai Institutes for Biological Sciences (SIBS). The human LSCC cell line NCI-H1703 was obtained from Guangzhou Cellcook Biotech Co., Ltd. The human LSCC cell line SK-MES-1 was obtained from Hangzhou Young Eagle Biotechnologe Co., Ltd. The HFL1 cell line was cultured in Ham’s F12K (Kaighn’s) Medium (Gibco 21127022, Thermo Fisher, Scotland, UK). NCI-H226, NCI-H1703 and SK-MES-1 cells were cultured in RPMI 1640 (GIBCO A1049101, Thermo Fisher, Grand Island, NY, USA). All media were supplemented with 10% fetal bovine serum (Clark FB25011, Richnond, USA) and 1% Penicillin–Streptomycin (GIBCO 10378016, Thermo Fisher, Waltham, MA, USA). All cells were maintained in the corresponding media at 37 ℃ with a humidified atmosphere of 5% CO_2_.

### Q-PCR

Total RNA from NCI-H1703 cells (shEIF4G1-vector and shEIF4G1-KD) was extracted with TRIzol^™^ (Invitrogen) and reverse transcription was performed using a first-strand cDNA Synthesis Kit (Takara) according to the manufacturer's instructions. Real-time polymerase chain reaction (RT-PCR) was performed using Premix Ex Taq SYBR Green PCR (Takara) on an ABI PRISM 7500 (Applied Biosystems) according to the manufacturer's instructions. The sequences of the primers were as follows: EIF4G1 forward, 5′-TTGTGGATGATGGTGGCT-3′ and reverse, 5′-TTA TCTGTGCTTTCTGTGGGT-3′; β-actin forward, 5′-CTCCATCCTGGCC TCGCT-3′, and reverse, 5′-GCTGTCACCTTCACCGTTCC-3′. β-actin served as the internal control. The expression levels of EIF4G1 were standardized according to the expression levels of β-actin and those with the value between 3/2 and 2/3 were defined as stable.

### Transfection

NCI-H1703, NCI-H226 and SK-MES-1 cells were plated in 60 mm dishes or 6-well plates. The cells were cultured for 24 h and the medium was replaced by Opti-MEM (Invitrogen) without Penicillin–Streptomycin. The siRNA sequences against EIF4G1 were the following: (1) 5'-GGAUCCCACUAGACUACAATT-3'; (2) 5'-GAUGUUAACAGAGG CAAUATT-3'. (3) The negative control sequences used were as follows: 5’-UUCUCCGAACGUGUCAC GUTT-3’. A total of 83 pmol siRNA (GenePharma, Shanghai, China) and 7.5 μl lipofectamine 3000 (Invitrogen) were incubated and added to the cells. The incubations were performed in 6-well plates. Following 48 h of incubation, the cells were collected for western blot analysis.

### Western blotting

NCI-H1703, NCI-H226 and SK-MES-1 cells were collected following transfection with siRNA-EIF4G1. The cells were lysed using RIPA (EpiZyme) with protease inhibitor cocktails (Bimake). A total of 30 μg of cell lysate was added to 6–20% SDS-PAGE gels and the proteins were transferred to 0.45 mm PVDF membranes (PerkinElmer Life Sciences, Inc.). The membranes were blocked in TBST with 5% BSA for 2 h at room temperature and they were subsequently incubated with primary antibodies against EIF4G1 (Cell Signaling Technology #8701, 1:1,000), AKT (Immunoway #YP0007, 1:1,000), p-AKT (ABclonal technology #AP1208, 1:1,000), mTOR (Bioss Anti Bodies #bs3494R, 1:1,000), cyclin D1 (ABclonal technology #A11022, 1:1,000), β-actin (Cell Signaling Technology, 1:1,000) at 4 ℃ overnight (more than 12 h). HRP (Invitrogen) was conjugated to a secondary antibody which was incubated at room temperature for 1 h with the membranes. Enhanced chemiluminescence reaction (Beyotime Institute of Biotechnology) was used to identify protein bands, which were visualized using autoradiography.

### Cell proliferation assay

NCI-H1703 and NCI-H226 cells were seeded at a density of 5000 cells/well in 96-well plates allowed to proliferate for 16–24 h and transfected with siRNA-EIF4G1 or Negative Control. Subsequently, 10 μl Cell Counting Kit-8 reagent (CCK-8, KeyGEN BioTECH, Nanjing, China) was added into each well at 0, 24, 48 and 72 h following transfection. The plates were incubated for 2 h at 37 ℃ and the optical density (OD) was measured at 450 nm using a microplate reader (SpectraMax M5, Molecular Devices, USA).

### Colony formation assay

Approximately 5000 NCI-H1703 and/or NCI-H226 cells were seeded to each well in 6-well plates. Following 2 weeks of incubation, the cells were fixed with 4% paraformaldehyde (20 min fixation), washed twice with PBS and stained with 0.1% Crystal Violet. The number of colonies containing ≥ 50 cells was counted under a microscope. The colony formation efficiency was calculated as follows: efficiency = (number of colonies/number of cells inoculated) × 100%. Each experiment was performed in triplicate.

### Flow cytometry

NCI-H1703 and NCI-H226 cells were serum-starved for 24 h and transfected with siRNA for 24 h. The cells were collected and washed twice with cold PBS. These cells were precipitated, mixed with 70% cold ethanol (− 20 ℃), resuspended in PBS, stained with propidium iodide (BD Pharmingen 556463) containing RNase A, incubated for 30 min at room temperature and analyzed by flow cytometry. Each assay was repeated at least three times.

### Xenograft model

Female BALB/C mice (5 weeks old) were purchased from SHANGHAI SLRC LABORATORY ANIMAL CO. LTD. The animals were allowed one week of adaptive feeding in SPF-class housing of laboratory. For the H1703 xenograft study, BALB/C mice were implanted subcutaneously at the middle or posterior armpit with 6 × 10^6^ cells diluted in PBS (*V*/*V* = 1:1). The tumor volume was monitored once every 2 days for the shEIF4G1-vector and shEIF4G1-KD groups. The experiments were terminated when the tumor volume was approximately 2500 mm^3^, and 10% Chloral hydrate (Sangon Biotech (Shanghai) Co., Ltd.) was administered intraperitoneally at a dose of 0.01 ml/g for 3 min, then the cervical vertebrae was dislocated. The tumor volume was calculated as 1/2 × *L* × *W*^2^ (*L* and *W* are the length and width of the tumor, respectively).

### Statistical analysis

The enumeration data, the expression levels of EIF4G1 and the assessment of cell proliferation were described by the mean ± SD. The degree of invasion and tumorigenesis in vivo were described by the mean ± SEM. The Student’s *t* test was performed for two group comparison, whereas one-way ANOVA was performed for multiple data comparison. The Mann–Whitney *U* test was used to analyze the association between the expression levels of EIF4G1 and the clinicopathological characteristics. The GraphPadPrism 5 software was used for graphical presentation. The prognostic analysis was performed using the multivariate Cox proportional hazards regression analysis. Univariate and multivariate Cox regression analyses were performed for estimation of disease prognosis. The Kaplan–Meier curve was used for survival curve estimation. All data were analyzed with SPSS 17.0 (IBM Corporation, Armonk) and a *P* value < 0.05 was considered for significant differences.

## Results

### EIF4G1 is highly expressed in LSCC tumor tissues and cell lines

**Fig. 1:**
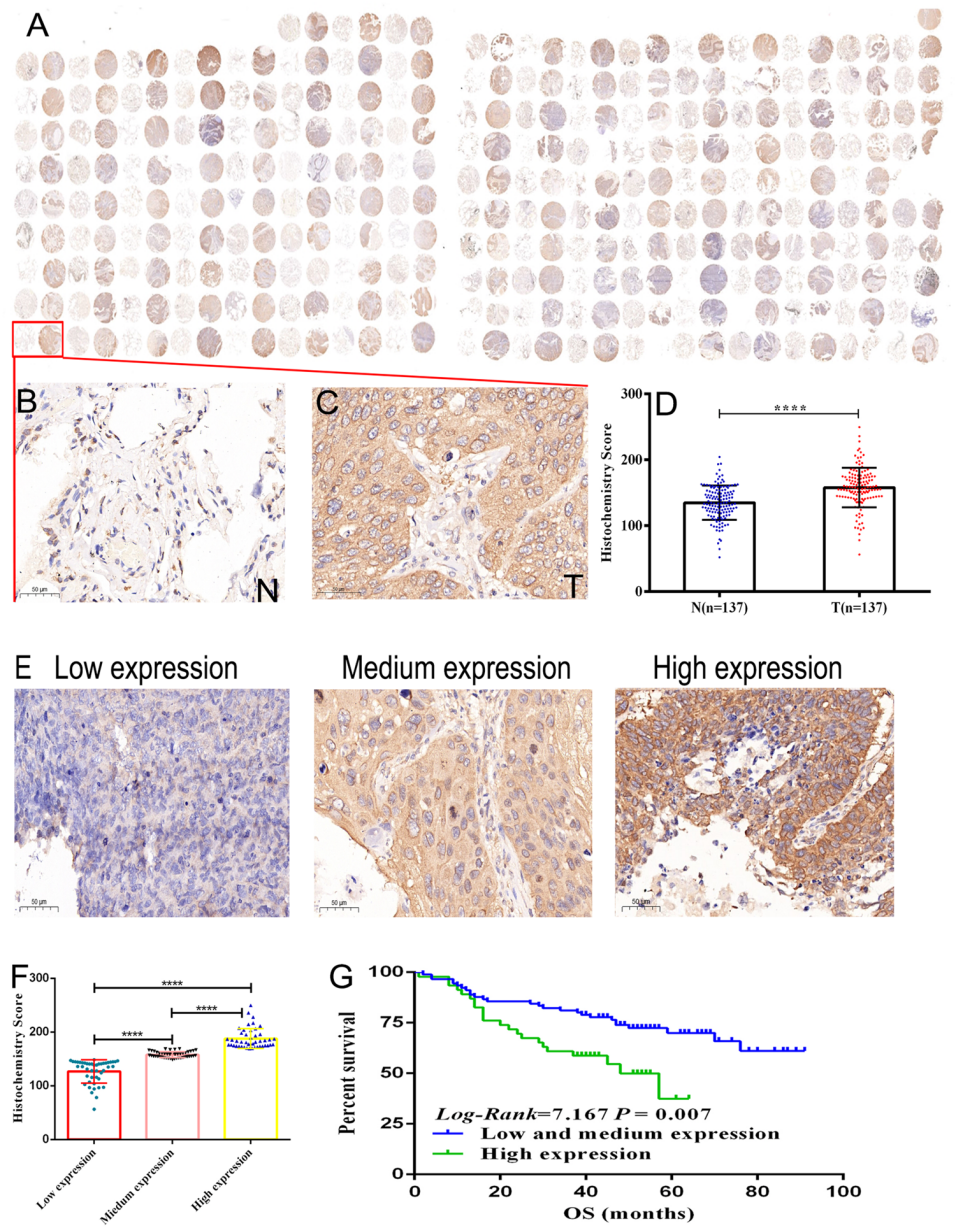
High expression of EIF4G1 in LSCC tumor tissues. **A** Tissue array, adjacent tissues (N) are shown on the left, while tumor tissues (T) are shown on the right by immunohistochemical staining. **B** The expression levels of EIF4G1 in adjacent tissues (magnification 400X). **C**, **D** The expression of EIF4G1 in tumor tissues (magnification 400X), H-score of adjacent and tumor tissues (*N* = 137). **E**, **F** The differences in H-score among low, medium and high expression of EIF4G1 in tumor tissues (magnification 400X). **G** Kaplan–Meier survival analysis of overall survival in 137 LSCC patients based on the levels of EIF4G1 protein expression. The IHC score was tested. Error bars represent the SD. ****P < 0.001. These were repeated three times

**Fig. 2:**
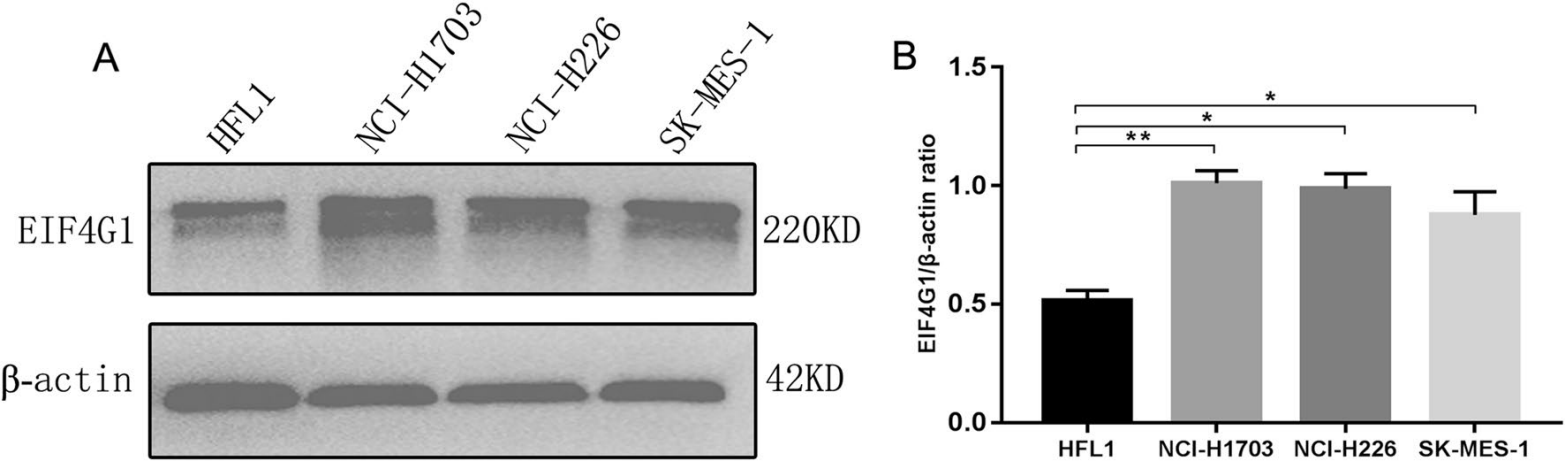
EIF4G1 is overexpressed in LSCC cell lines. **A** Western blot analysis indicated that HFL1 had a lower expression of EIF4G1 than LSCC. **B** β-actin was used as the internal control, EIF4G1/β-actin ratio indicated significant differences between HFL1 and LSCC. The error bars represent the SD. *P < 0.05, **P < 0.01.These were repeated three times

Initially, EIF4G1 protein expression was examined in 165 paired LSCC patient tissues. A total of 137 paired LSCC patient tissues were analyzed by IHC (Fig. 1A). The analysis of LSCC adjacent (Fig. 1B) and paired tumor (Fig. 1C) tissues indicated that the expression levels of EIF4G1 in LSCC tumor tissues were higher than those of the adjacent tissues (Fig. 1D). We further assessed the expression levels of EIF4G1 according to three subgroups (Fig. 1E) and the data demonstrated significant differences (Fig. 1F). Furthermore, we found that EIF4G1 expression was markedly increased in LSCC cell lines (NCI-H1703, NCI-H226 and SK-MES-1) compared to that of the normal human fetal lung cell line HFL1 (Fig. 2A, B).

Subsequently, the prognostic value of EIF4G1 protein expression was assessed by Kaplan–Meier plotter analysis. The Kaplan-Meir plots of overall survival (OS; Fig. 1G) indicated that high expression levels of EIF4G1 were associated with poor survival (*P* = 0.007). These findings suggested that EIF4G1 overexpression could predict poor survival in LSCC.

### Association between clinicopathological characteristics and EIF4G1 expression in patients with LSCC

The parameters sex and pathological grade were not significantly associated with EIF4G1 expression in 137 patients with LSCC. However, age (*P* ˂ 0.001) and clinical stage (I vs. II vs. III + IV) (*P* ˂ 0.001) were significantly associated with the expression of EIF4G1 (Table 1).

**Table 1:** Expression and clinicopathological features of EIF4G1 protein in LSCC

Characteristcs	EIF4G1 protein expression			
	*N*	Low and medium (*N*)	High (N)	*P* value
Age	137	91	46	< 0.001
Sex				
Male	129	86	43	0.74
Female	8	5	3	
Clinical Stage				
I	51	39	12	< 0.001
II	60	39	21	
III + IV	26	13	13	
Pathological grade				
1	14	10	4	0.463
2	110	73	37	
3	13	8	5	

To investigate the prognostic value of EIF4G1 in LSCC, the association between the expression levels of EIF4G1 and patient survival was assessed in 137 LSCC cases. Patients with high expression of EIF4G1 had significantly lower overall survival time (*P* = 0.007) (Fig. 1G). To estimate the clinical significance of various prognostic factors that may influence the survival and tumor progression in LSCC patients, univariate analysis was performed. As summarized in Table 2, the parameters age (*P* = 0.002), clinical stage (I vs. II vs. III + IV) (*P* < 0.001) and high expression of EIF4G1 (*P* = 0.009) were significant risk factors affecting the overall survival of LSCC patients. To determine the independent prognostic effects of these variables, multivariate analyses were performed using the Cox’s proportional hazard model. The results indicated that age (*P* < 0.001), clinical stage (*P* < 0.001) and high expression of EIF4G1 (*P* = 0.008) could predict the overall survival of the patients (Table 2). Taken together, the findings indicated that the expression levels of EIF4G1 could serve as an effective biomarker for the prognosis of LSCC patients.

**Table 2:** Summary of univariate and multivariate Cox regession analysis of overall survival duration

Parameter	Univariate analysis			Multivariate analysis		
	*P*	HR	95%CI	*P*	HR	95%CI
Age	0.002	1.055	1.020–1.092	< 0.001	1.069	1.030–1.109
Sex						
Male vs. female	0.940	1.044	0.324–3.373			
Clinical Stage						
I vs. II vs. III + IV	< 0.001	1.637	1.315–2.038	< 0.001	1.6	1.285–1.991
Pathological grade						
1 vs.2 vs. 3	0.232	0.688	0.373–1.271			
EIF4G1 expression high expression vs. low and medium expression	0.009	2.155	1.210–3.839	0.008	2.255	1.238–4.105

### EIF4G1 promotes the proliferation of LSCC cells

**Fig. 3:**
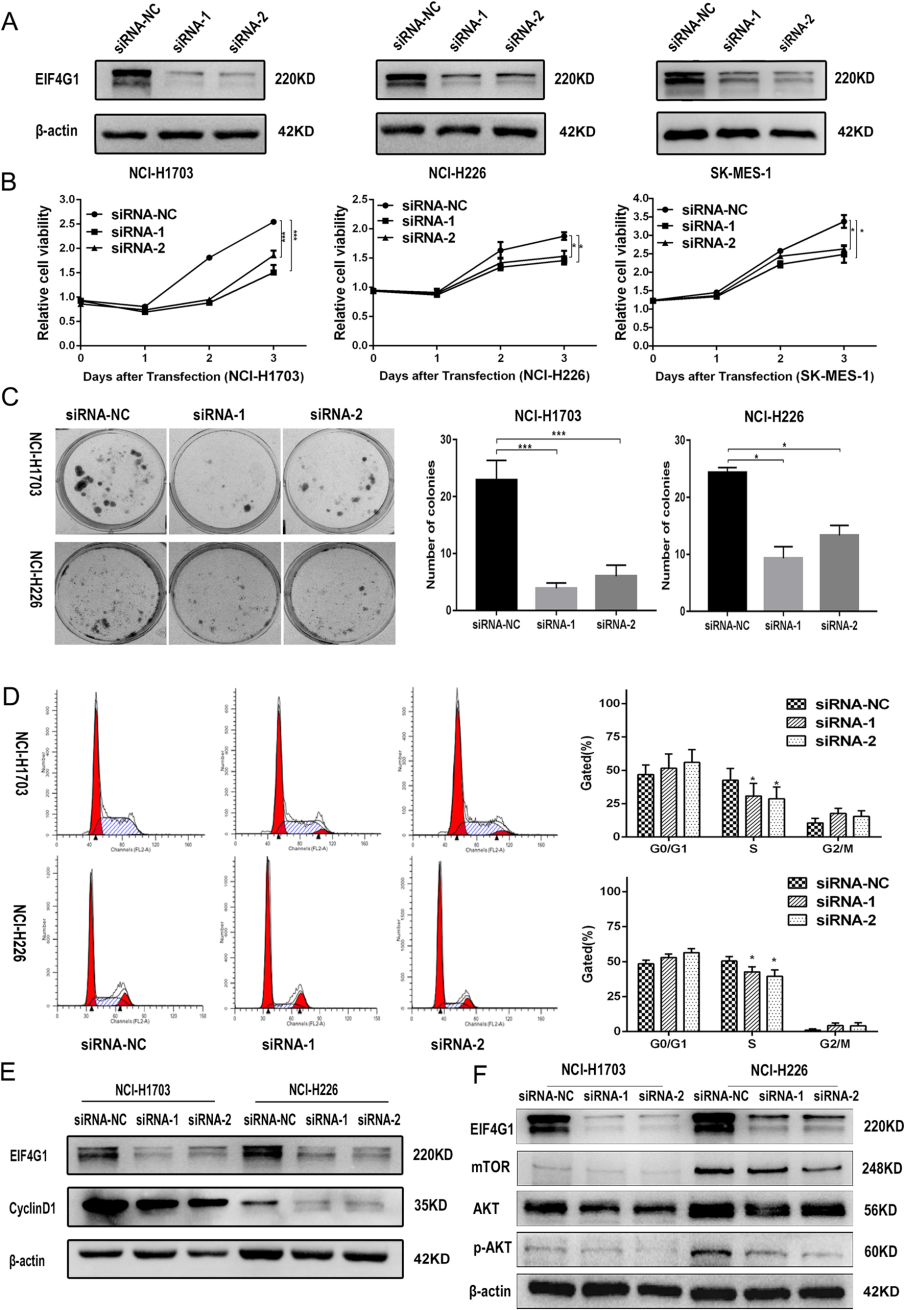
EIF4G1 affects the proliferation in LSCC cells. **A** Western blot analysis indicated an effective decrease in protein expression of EIF4G1 using siRNA-1&2 compared to the expression noted in the siRNA-NC group. **B** The cell growth of transient EIF4G1 “knockdown” LSCC cells and control (siRNA-NC) was measured and compared by the CCK-8 assay. **C** The anchorage-independent growth abilities of transient EIF4G1 “knockdown” NCI-H1703 & NCI-H226 cells and control (siRNA-NC) were measured by the colony formation assay. **D** The cell cycle was determined by PI staining and flow cytometry analysis, whereas protein expression was detected by western blotting. **E**, **F** The transient EIF4G1 “knockdown” was performed for 48 h and the samples were analyzed with regard to their protein levels. The error bars represented the SD. *P < 0.05, **P < 0.01, ***P < 0.005. These were repeated three times

To examine the effects of EIF4G1 on cell proliferation, RNAi was used to knockdown the expression of EIF4G1 in NCI-H1703, NCI-H226 and SK-MES-1 cells and to subsequently examine cell proliferation. We confirmed that RNAi (siRNA-1, siRNA-2) successfully reduced EIF4G1 expression (Fig. 3A). Furthermore, downregulation of EIF4G1 effectively reduced LSCC cell proliferation as determined by the CCK-8 proliferation assay (Fig. 3B). Subsequently, the data demonstrated that EIF4G1 knockdown effectively inhibited LSCC colony formation (Fig. 3C).

To further assess the mechanisms of EIF4G1-mediated LSCC cell growth regulation, the LSCC cell cycle was analyzed using flow cytometry. The analysis indicated that EIF4G1 dowregulation induced G1 cell cycle arrest in NCI-H1703 and NCI-H226 (Fig. 3D, E) cell lines. Furthermore, EIF4G1 decreased cyclinD1 expression (Fig. 3F). To further investigate the molecular mechanisms of EIF4G1 regulation of LSCC cell proliferation, the activity levels of mTOR were assessed by western blot analysis. Downregulation of EIF4G1 reduced mTOR, AKT2 and p-AKT levels (Fig. 3G). Taken together the data indicated that EIF4G1 may regulate LSCC cell proliferation by targeting mTOR and the CyclinD1 signaling pathways.

### *EIF4G1 regulates LSCC tumorigenesis *in vivo

**Fig. 4:**
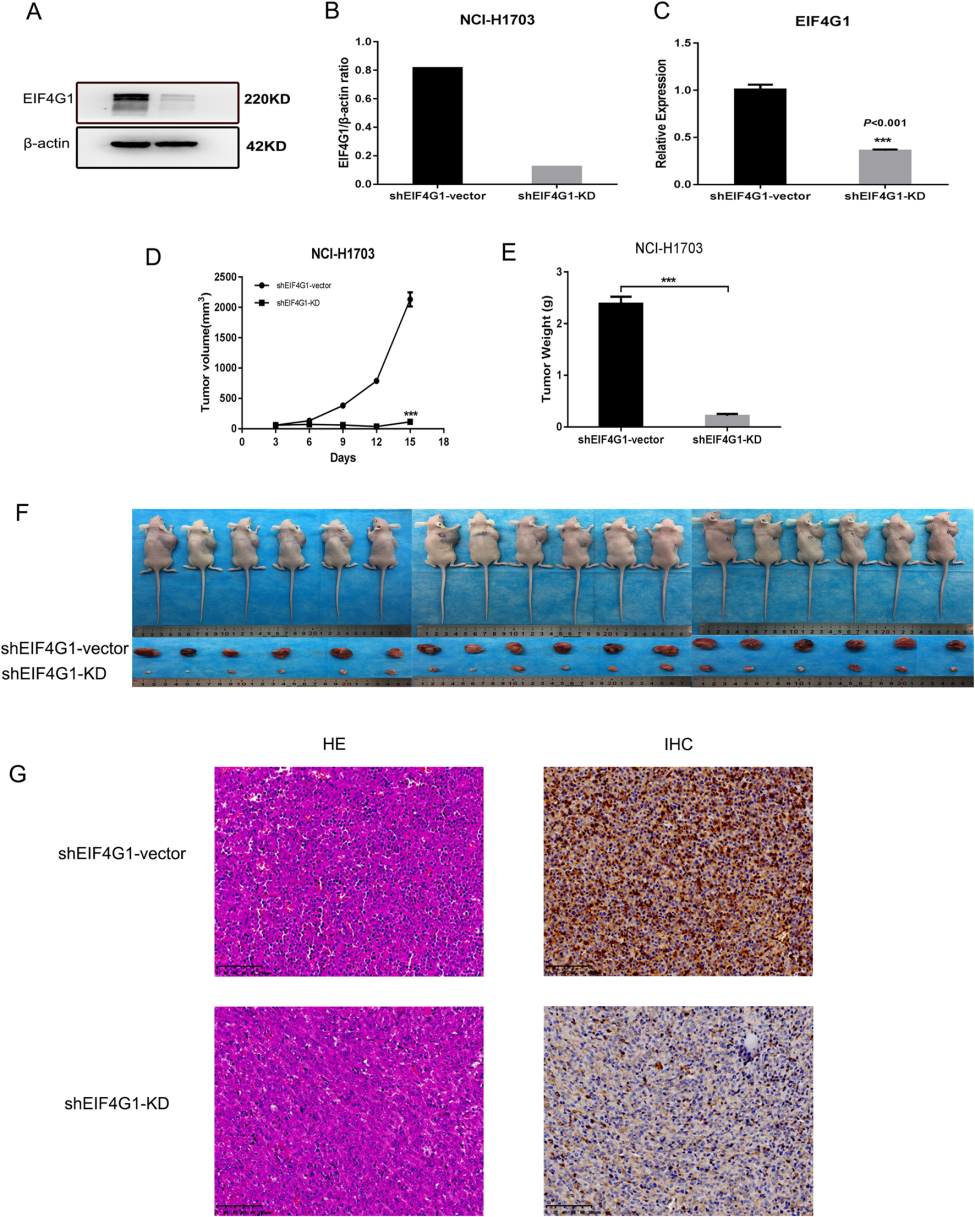
EIF4G1 regulates LSCC tumorigenesis in vivo **A**, **B**. The expression levels of EIF4G1 in stable “knockdown” NCI-H1703 cells was performed using lentiviral vectors containing 2 shRNA specifically targeting EIF4G1. The protein expression was detected by western blot analysis. A non-silence (vector)-shRNA was used as a negative control. **C** A Q-PCR was used to measure the expression of EIF4G1 in tumors from BLAB/C mice. ***P < 0.005. **D **NCI-H1703 cells containing shEIF4G1-vector or shEIF4G1-KD lentiviral vectors were injected into nude mice (*n* = 18 for each group). The tumor volume was recorded every three days for a maximum period of 15 days. The data are presented as mean ± SEM. ***P < 0.005. **E** The tumors were harvested and weighted. The data are presented as mean ± SEM. ***P < 0.005. **F** The volume and weight were compared between the shEIF4G1-vector and shEIF4G1-KD **G**. **H**, **E** staining indicated cellular morphology. EIF4G1 protein expression was markedly decreased in xenograft tumors of the shEIF4G1-KD group compared with that noted in the shRNA-vector group

To assess the role of EIF4G1 in LSCC tumorigenesis in vivo, the xenograft model was established by injecting vector or EIF4G1 stable knockdown NCI-H1703 cells. In the present study, the shEIF4G1-KD group demonstrated reduced EIF4G1 levels by western blot (Fig. 4A, B) and Q-PCR analyses (Fig. 4C). Depletion of EIF4G1 significantly decreased tumor growth in vivo compared with that noted in the vector cells (Fig. 4D, F). Concomitantly, immunohistochemical analysis was used to determine cell morphology and the results indicated that the shEIF4G1-KD group exhibited significantly decreased EIF4G1 levels than the shEIF4G1-vector group (Fig. 4G).

## Discussion

Our research finds EIF4G1 promotes LSCC cell proliferation and may represent an indicator of prognosis in LSCC.

The current study indicated that EIF4G1 expression was highly expressed in LSCC tissues and cells, which was in agreement with the data reported by Bauer et al. [15] EIF4G1 can predict LSCC prognosis and regulate the mTOR/AKT and Cyclin D1 signaling pathways to promote proliferation and invasion in LSCC.

The study conducted by Bauer et al. [15] reported that EIF4G1 expression was upregulated and was not associated with TNM in LSCC, which is in agreement with the data reported in the present study. In addition, EIF4G1 was shown to predict LSCC prognosis. The study conducted by Jaiswal et al. [5] demonstrated that EIF4G1 could predict the prognosis of patients with nine different types of cancer, with the exception of skin cutaneous melanoma. EIF4G1 is a member of the EIF4G family. In head and neck cancer, the mutation of EIF4G1 affects the activation of mTOR signaling [18]. SBI-756 and 4EGI-1 can inhibit the activity levels of EIF4G1 and the activation of the AKT pathway [12]. When sufficient nutrients are available, FRAP/mTOR transmits a positive signal to the S6Ks and certain kinase enzymes. X may phosphorylate eIF4GI [19], which increases the activity levels of EIF4G1. EIF4G1 can be activated by the AKT/mTOR pathway. These data do not explain whether EIF4G1 can effect AKT and mTOR in LSCC. The current study demonstrated that EIF4G1 could effect AKT and mTOR in LSCC. Future studies are required to confirm the exact interaction between EIF4G1 and AKT/mTOR.

Immunoblotting and Q-PCR analyses indicated that EIF4G1 expression was lower in the downregulated group than that noted in the empty vector group and that downregulation of EIF4G1 could significantly inhibit the proliferation of LSCC in vivo. The volume, quality, H&E staining and immunohistochemical profiles of the tumors in the nude mice were consistent with the results of the in vitro experiments. The results reported by Tu et al. [20] were consistent with those reported in the current experiment, which indicated that EIF4G1 could exert optimal proliferative ability in these two types of cancer.

In conclusion, the present study indicated that EIF4G1 was overexpressed in LSCC and that it could predict disease prognosis. It also regulated cyclin D levels and cell proliferation via the AKT/mTOR pathway in LSCC.

## Data Availability

All data are fully available.
